# Effect of ionizing radiation on the mechanical properties of current fluoride-releasing materials

**DOI:** 10.1038/s41405-024-00192-w

**Published:** 2024-02-19

**Authors:** Pimduean Sivavong, Chanyared Sanprasert, Proudfah Leekhaphan, Somsuda Chooboonlarp, Chalermchart Bunsong, Chawalid Pianmee, Potsawat Poolkerd, Thawanrat Singthong, Puliwan Gorwong, Dusit Nantanapiboon

**Affiliations:** 1 Department of Operative Dentistry, Faculty of Dentistry, Chulalongkorn University, Bangkok, 10330 Thailand; 2 Faculty of Dentistry, Chulalongkorn University, Bangkok, 10330 Thailand; 3 Department of Radiation Therapy, Chonburi Cancer Hospital, Chonburi, 20000 Thailand; 4 Dental Department, Surin Hospital, Surin, 32000 Thailand; 5 Dental Department, Panyananthaphikkhu Chonprathan Medical Center, Srinakharinwirot University, Nonthaburi, 11120 Thailand; 6 Dental Material Research and Development Center, Faculty of Dentistry, Chulalongkorn University, Bangkok, 10330 Thailand

**Keywords:** Glass-ionomer cement, Gerodontics

## Abstract

**Objectives:**

This study aimed to evaluate the effect of fractional radiation on the mechanical properties of fluoride-releasing materials.

**Materials and methods:**

High-viscosity glass ionomer cement (F9), resin-modified glass ionomer cement (F2), glass hybrid restoration (EQ), and bioactive composite (AC) were divided into 3 subgroups: 0, 35, and 70 Gy fractional radiation doses. The specimens were subjected to surface roughness, Vickers microhardness, and compressive strength tests. The chemical components and morphology of the tested specimens were observed via energy dispersive spectroscopy and scanning electron microscopy. The data were analyzed using two–way ANOVA with Bonferroni post hoc analysis.

**Results:**

After exposure to fractional radiation, the surface roughness increased in all the groups. F9 had the highest surface roughness, while AC had the lowest surface roughness within the same radiation dose. The Vickers microhardness decreased in F9 and EQ. The AC had the highest compressive strength among all the groups, followed by F2. More cracks and voids were inspected, and no substantial differences in the chemical components were observed.

**Conclusions:**

After fractional radiation, the surface roughness of all fluoride-releasing materials increased, while the Vickers microhardness of F9 and EQ decreased. However, the compressive strength increased only in F2 and AC.

## Introduction

Head and neck cancer is a significant global health challenge. The newly diagnosed cases annually comprise approximately 380,000 in the lip and oral cavity, 133,000 in the nasopharynx, 98,000 in the oropharynx, and 54,000 in the salivary glands [1]. Among the primary treatment modalities for head and neck cancer patients, radiotherapy is commonly employed. Its impact extends beyond the intended target of malignant tumors, affecting adjacent tissues and dental restorative materials. A multitude of complications can arise as a consequence of radiation exposure after head and neck radiotherapy (HNRT) since radiation can damage salivary glands, causing a lack of saliva or xerostomia, which leads to radiation caries [2].

Fluoride-releasing materials such as glass ionomer cement (GIC) have been widely used in restorative dentistry to treat carious lesions for several decades and have been developed to improve their mechanical properties. The development of fluoride-releasing materials has focused mainly on increasing the amount of glass filler and liquid. High viscosity GICs (Fuji IX GP Extra - F9) are categorized as conventional GICs since they are produced by acid-base reactions from powder – liquid mixtures with higher powder/liquid ratios and high molecular weight polyacrylic acid [3, 4]. To improve the properties of conventional GIC, resin monomers which are 2-hydroxyethyl methacrylate (HEMA) and urethane dimethacrylate (UDMA), along with photoinitiators such as camphorquinone have been incorporated into RMGIC (Fuji II LC – F2). RMGIC still retains the acid–base reaction of conventional GIC with the addition of radical polymerization of the methacrylate monomer. Another development of fluoride - releasing materials is the use of a glass hybrid material (Equia Forte - EQ) based on GIC technology. The conventional glass matrix was reinforced with ultrafine and highly reactive glass particles dispersed in a conventional glass ionomer structure and included high molecular weight polyacrylic acid [5]. Subsequently, a new bioactive composite (ACTIVA BioACTIVE–RESTORATIVE – AC) has been introduced into the market. The material contains high molecular weight polyacrylic acid similar to high viscosity GIC and RMGIC with the addition of urethane dimethacrylate monomers and dimethacrylate phosphate. All of these fluoride-releasing materials mentioned above are commonly used for both anterior and posterior direct restorations in HNRT patients due to their ability to prevent caries progression before the initiation of radiotherapy, which is particularly important for maintaining oral health in individuals undergoing treatment for head and neck cancer.

An in vitro study revealed that ionizing radiation directly affected the mechanical properties of fluoride-releasing materials. Although several studies stated that radiation can alter the properties of restorative materials, such as microhardness, surface roughness, and compressive strength [3, 6, 7], some studies have shown no detrimental effects [8–10]. The scientific literature presents a degree of controversy in regard to the interaction between ionizing radiation and fluoride-releasing materials. Additionally, there is currently limited or no literature on the impact of radiation on these new fluoride-releasing materials.

Owing to the lack of scientific evidence, this study aimed to evaluate the effect of radiation on the mechanical properties of fluoride-releasing materials, including high viscosity GIC, RMGIC, glass hybrid restoration and bioactive composite. The null hypothesis was that radiation would not alter the mechanical properties of fluoride-releasing materials.

## Materials and methods

Ethical review and approval were waived for this study due to the nature of study was the material study. There was no involvement of human subjects and human specimens. Four fluoride-releasing materials were used in this study. Table 1 presents the composition, manufacturer’s instructions, and group codes of tested materials. The specimens in each group were divided into 3 subgroups: nonirradiated (control group) and irradiated with 35 Gy, and 70 Gy (experimental group).

**Table 1:** Composition, manufacturer’s instructions, and group codes of the materials.

Materials	Composition	Manufacturer’s instruction	Group code
High - viscosity GIC (GC Fuji IX GC Extra, Tokyo, Japan); F9 Lot #2102192	Powder: Fluoroaluminosilicate glass, polyacrylic acid powder Liquid: polyacrylic acid, polybasic carboxylic acid	1. Capsule activation and mixing a) Before activation, shake the capsule or tap its side on a hard surface to loosen the powder. b) To activate the capsule, push the plunger until it is flush with the main body. c) Immediately place the capsule into a metal GC Capsule Applier and click the lever once. d) Immediately remove the capsule and set it into a mixer and mix for 10 s. 2. Restorative technique a) Immediately remove the mixed capsule from the mixer and load it into the GC Capsule Applier. b) Make two clicks to prime the capsule then syringe. The working time is 1 min 15 s from start of mixing at 23 °C (73.4 °F). Higher temperatures will shorten working time. c) Within 10 s maximum after mixing, start to extrude the mixture directly into the preparation. d) Form the preliminary contour, and cover with a matrix if required. 3. Finishing a) Final finishing under water spray using standard techniques can begin approximately 2 min 30 s after start of mixing.	F9-0 (Non-Irradiated)
			F9-35 (Irradiated 35 Gy)
			F9-70 (Irradiated 70 Gy)
RMGIC (GC Fuji II LC capsules, Tokyo, Japan); F2 Lot #2102111	Powder: Aluminofluorosilicate glass Liquid: Polyacrylic acid, tartaric acid, distilled water, camphorquinone, dibutyl hydroxy toluene, and three resin complexes (mainly HEMA)	1. Capsule activation and mixing a) Before activation, shake the capsule or tap its side on hard surface to loosen the powder. b) To activate the capsule, push the plunger until it is flush with the main body and hold it down for 2 s. c) Immediately removed the capsule and set it into a mixer and mix for 10 s at high speed. 2. Restorative technique a) Immediately remove the mixed capsule from the mixer and load it into the GC Capsule Applier. b) Click twice to prime the capsule then syringe. c) Extrude cement directly into preparation. Avoid air bubbles. d) Light - cure for 20 s. 3. Finishing a) Finishing under water spray.	F2-0 (Non-Irradiated)
			F2-35 (Irradiated 35 Gy)
			F2-70 (Irradiated 70 Gy)
Glass hybrid restoration (Equia Forte^TM^, GC, Tokyo, Japan); EQ Lot #2108031	Powder: 95% strontium-fluoroaluminosilicate glass, 5% polyacrylic acid Liquid: 40% aqueous polyacrylic acid	1. Capsule activation and mixing a) Before activation, shake the capsule or tap its side on a hard surface to loosen the powder. b) To activate the capsule, push the plunger until it is flush with the main body and hold it down for 2 s. c) Immediately remove the capsule and set it into a mixer and mix for 10 s. 2. Restorative technique a) Immediately remove the mixed capsule from the mixer and load it into a GC capsule applier. b) Click twice to prime the capsule. c) Within 10 s maximum after mixing, start to extrude the mixture directly into preparation. 3. Finishing a) Finishing under water spray using superfine diamond burs after 2 min 30 s from start of mixing.	EQ-0 (Non-Irradiated)
			EQ-35 (Irradiated 35 Gy)
			EQ-70 (Irradiated 70 Gy)
Bioactive composite (ACTIVA^TM^ BioACTIVE –RESTORATIVE, Pulpdent, Watertown, MA, USA); AC Lot #211206	Powder: silanated bioactive glass and calcium, silanated silica and sodium fluoride Liquid: mix of methacrylates and diurethane with modified polyacrylic acid, and water	1. Set curing light to 20-s, low intensity setting. 2. Apply a bonding agent of your choice. 3. Place a mix tip on the ACTIVA syringe. Insert syringe into ACTIVA-SPENSER and snap into place using firm pressure. 4. Dispense material using gentle pressure. To ensure an even mix of base and catalyst, dispense 1–2 mm of material onto a mixing pad and discard this material. 5. Place mix tip at cavity floor. Apply ACTIVA in increments of up to 4 mm, keeping mix tip submerged in the material. Light cure for 20 s between each layer. Finishing and polishing in the usual manner.	AC-0 (Non-Irradiated)
			AC-35 (Irradiated 35 Gy)
			AC-70 (Irradiated 70 Gy)

### Sample size calculation

The sample size was calculated using G*Power software version 3.1. The parameters were set with a 95% confidence interval, 95% power, and 0.97 effect size. The reference values were taken from a previous study by Brandeburski et al. [11]. The total sample size was calculated as at least seven specimens per group. Therefore, this study was designed to use 10 specimens per group.

### Specimen preparation for the surface roughness and microhardness test

The specimens for F9 and EQ were prepared in transparent cylindrical acrylic molds 4 mm in diameter and 2 mm in height. The capsule was mixed according to the manufacturer’s instructions. The capsule was inserted into a capsule applier, dispensed into the molds avoiding air bubble entrapment and covered with microscope coverslips. The specimens were left for the completed set.

The specimens for F2 and AC were prepared in transparent cylindrical acrylic molds 4 mm in diameter and 2 mm in height. The capsule was mixed according to the manufacturer’s instructions. The capsule was inserted into a capsule applier, dispensed into transparent cylindrical acrylic molds avoiding air bubble entrapment and covered with microscope coverslips. Then, the specimen was light cured with a LED light - curing unit (1200 mW/mm^2^, Demi^TM^ Plus, Kerr, Orange, CA, USA) above the glass plate on both sides for 40 s to ensure complete polymerization of the materials.

All specimens were thoroughly measured with a digital vernier caliper to reassure the dimensions of the specimens, which were subsequently examined with a stereomicroscope at 10X magnification (Stereo Microscope SZ61, Olympus, Japan) to ensure that there were no gaps and voids.

### Specimen preparation for compressive strength test

The specimens for F9 and EQ were prepared in cylindrical metal molds 4 mm in diameter and 6 mm in height according to ISO9917-1 [12]. The capsule was mixed according to the manufacturer’s instructions. The capsule was inserted into a capsule applier, dispensed into the molds avoiding air bubble entrapment and covered with microscope coverslips.

The specimens for F2 and AC were prepared in cylindrical metal molds 4 mm in diameter and 6 mm in height according to ISO9917-2 [13]. The capsule was mixed according to the manufacturer’s instructions. The mold was filled with 2 mm increment of fluoride-releasing materials avoiding air bubble entrapment, and light cured for 40 s. Then, the next 2 mm increment was filled, and light cured for 40 s. The glass plate was placed above the final increment before light curing for 40 s. To ensure complete polymerization of the materials, the specimen was removed from the mold, and light-cured on the lateral surface area of the cylinder specimen for 40 s.

All specimens were placed on a sieve tray above deionized water in a plastic container at 37 ˚C with a relative humidity of 100%.

### Radiation procedure

After 24 h, the specimens in the experimental group were removed from the container and immersed in deionized water, blotted with paper, and exposed to 35 Gy and 70 Gy fractional-dose radiation according to the radiation dose for patients with low- and high-stage head and neck cancer, using an intensity-modulated irradiator (IMRT, Varian RapidArc, Varian Medical Systems, Palo Alto, CA, USA). In the irradiated 35 Gy group, the specimens were exposed to radiation at a rate of 2 Gy per day for 17 consecutive days, with an additional 1 Gy dose administered on the 18th day. For the 70 Gy irradiation group, the specimens received daily radiation doses of 2 Gy for a total of 35 days. The radiation procedure was performed by a radiation therapist at Chonburi Cancer Hospital, Chonburi, Thailand. To calibrate the radiation dose, the Eclipse program (Eclipse Veterinary Software Ltd., Great Chesterford, England) was used for calculation via a computerized tomography scan (Aquilion LB TSX-201A, Toshiba Corp., Tokyo, Japan). After radiation, the plastic containers were restored at 37 °C with a relative humidity of 100%. The radiation procedure was repeated until the assigned radiation dose was obtained for each group.

### Surface roughness and microhardness test

**Fig. 1:**
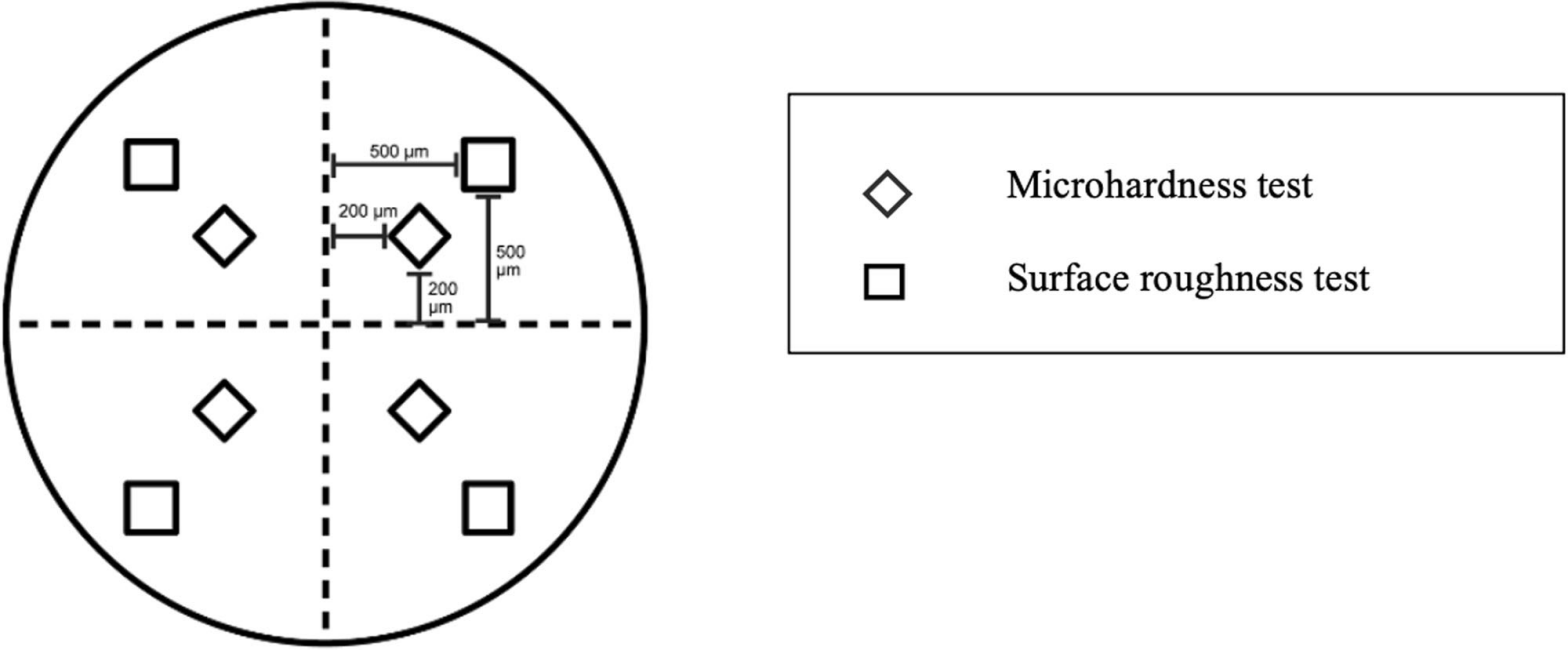
The location of the surface roughness and microhardness tests. Surface roughness and microhardness tests were conducted at distances of 500 and 200 microns horizontally and vertically from the reference lines, respectively.

Two lines were perpendicularly marked at the center of the specimen as reference lines shown in Fig. 1. The sites for conducting the surface roughness test were measured at a distance of 500 µm, while the sites for the microhardness test were measured at a distance of 200 µm from the horizontal and vertical lines, respectively.

The microhardness was analyzed using a Vickers microhardness tester (FM810, Future-Tech Corp., Kanagawa, Japan) with a 50 g load and 15 s dwell time. The mean microhardness of each specimen was obtained by calculating four indentations. After that, the surface roughness was assessed using non-contact profilometry with the Infinitefocus SL program (Alicona, Graz, Austria). A surface scan was performed with a 10x magnification lens in the area of 0.4 × 0.4 mm. The average roughness (Ra) from each image was calculated.

### Compressive strength test

All specimens were subjected to compressive strength test. The measurements were performed with a universal testing machine (8872, Instron, England) using a 2.5 cm long steel head. The load was applied at the center of the specimen with a 1 mm/min crosshead speed until the specimen fractured. The compressive strength of each specimen was expressed in MPa (N/mm^2^).

### Scanning electron microscopy (SEM) and energy dispersive spectroscopy (EDS)

**Fig. 2:**
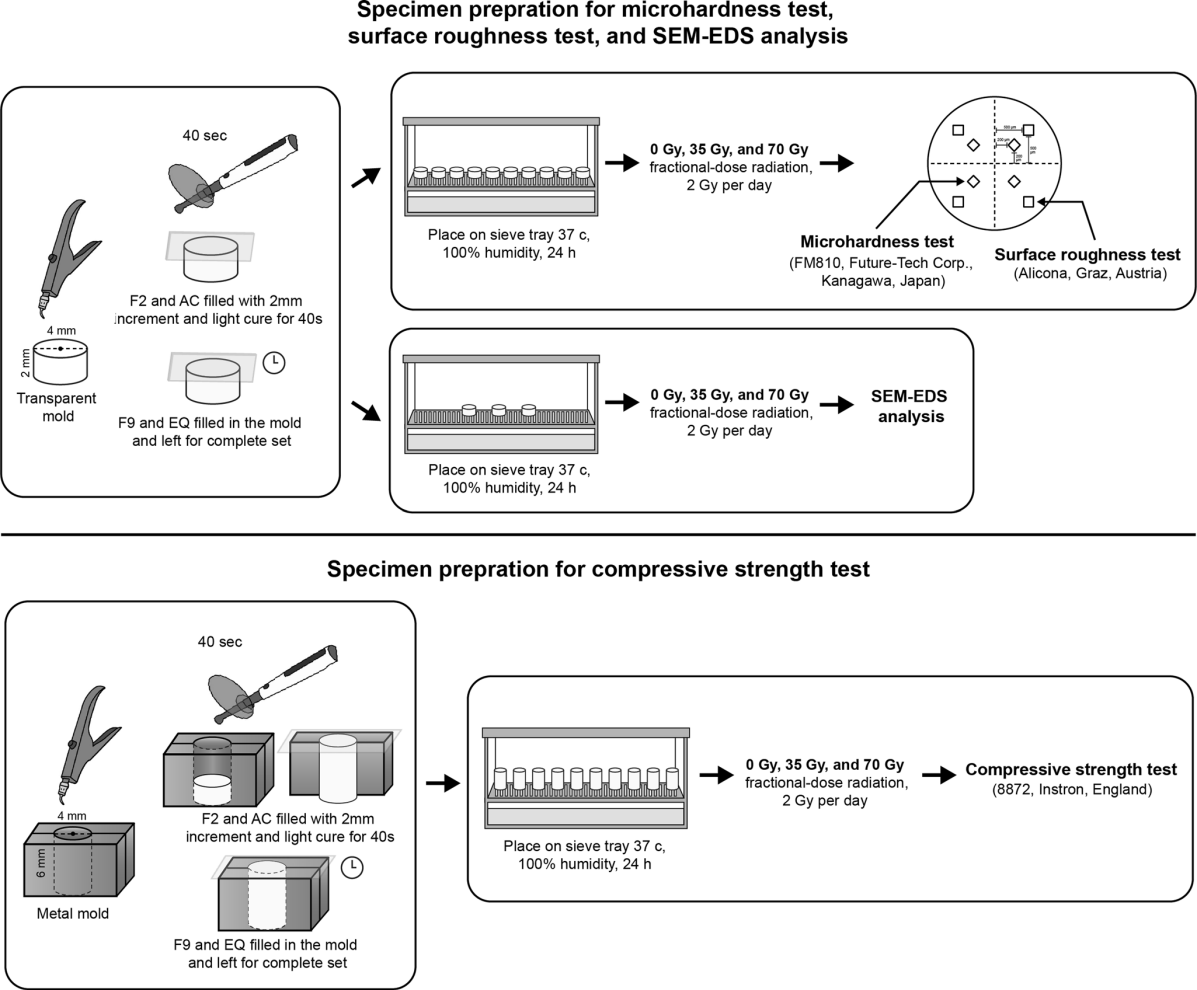
A flow chart representing the methodology used in this study. Upper part demonstrated the specimen preparation for microhardness test, surface roughness test, and SEM-EDS analysis while the lower part illustrated the specimen preparation for compressive strength test.

Three specimens were prepared using the same procedure as for specimen preparation for surface roughness test. The specimens in the experimental group were irradiated as described above. All specimens from each subgroup were coated with gold/palladium, and examined by SEM (Quanta250, FEI, USA). The SEM images were obtained at a magnification of 5000X. A point analysis was performed at the center of the specimens at 15 kV. The average distribution of the chemical compositions from the specimen in each subgroup was calculated as a relative percentage by weight (wt%). The methodology used in this study is shown in Fig. 2.

### Statistical analysis

All statistical procedures were performed using SPSS software (IBM SPSS statistics V29.0, IBM; Armonk, NY, USA). The data were evaluated for a normal distribution using the Shapiro-Wilk test. Two – way ANOVA was used to analyze the factors and their interactions. The microhardness, surface roughness and compressive strength were evaluated with Bonferroni post hoc analysis, for which the *p value* < 0.05. SEM - EDS analysis results were reported as descriptive statistics.

## Results

Two-way ANOVA revealed that type of fluoride-releasing materials and radiation dose significantly impacted the surface roughness, microhardness, and compressive strength, as did the interaction between these two factors, as shown in Table 2. The statistical analysis of the surface roughness, microhardness, and compressive strength is shown in Table 3.

**Table 2:** Two-way ANOVA for materials (A) and radiation doses (B) on surface roughness, Vickers microhardness, and compressive strength.

Tests	df	Sum of squares	Mean square	F	*p*-value
Surface roughness					
Materials (A)	3	349909.010	116636.337	145.012	<0.001
Radiation doses (B)	2	558942.107	279471.054	347.462	<0.001
A X B	6	131287.561	21881.260	27.205	<0.001
Microhardness					
Materials (A)	3	51891.849	17297.283	1447.783	<0.001
Radiation doses (B)	2	604.495	302.248	25.298	<0.001
A X B	6	633.837	105.640	8.842	<0.001
Compressive strength					
Materials (A)	3	329714.601	109904.867	361.531	<0.001
Radiation doses (B)	2	3259.115	1629.558	5.360	0.006
A X B	6	4140.822	690.137	2.270	0.042

**Table 3:** Mean (standard deviation) surface roughness (nm), Vickers microhardness (VHN), and compressive strength (MPa) at the different radiation doses.

Surface Roughness	0 Gy	35 Gy	70 Gy	*p*-value
F9	230.493^Aa^ ± (15.745)	419.415^Ab^ ± (43.291)	528.210^Ac^ ± (39.273)	<0.001
F2	224.587^Aa^ ± (14.285)	291.822^Bb^ ± (27.581)	330.186^Bc^ ± (26.626)	<0.001
EQ	229.805^Aa^ ± (18.170)	308.115^Bb^ ± (24.713)	377.499 ^Cc^ ± (35.221)	<0.001
AC	194.637^Ba^ ± (7.321)	233.095^Cb^ ± (15.410)	310.777^Bc^ ± (42.910)	<0.001
*p* value	<0.001	<0.001	<0.001	
Microhardness	0 Gy	35 Gy	70 Gy	*p*-value
F9	83.758^Aa^ ± (2.704)	75.656^Ab^ ± (3.606)	69.968^Ac^ ± (4.991)	<0.001
F2	64.381^Ba^ ± (3.616)	63.695^Ba^ ± (5.166)	63.388^Ba^ ± (3.880)	0.869
EQ	75.904^Ca^ ± (4.142)	72.568^Aab^ ± (3.422)	68.539^Ab^ ± (3.695)	0.001
AC	23.994 ^Da^ ± (0.410)	23.854^Ca^ ± (0.652)	24.202^Ca^ ± (0.458)	0.333
*p* value	<0.001	<0.001	<0.001	
Compressive strength	0 Gy	35 Gy	70 Gy	*p*-value
F9	110.681^Aa^ ± (19.297)	106.705^Aa^ ± (12.667)	108.643^Aa^ ± (13.433)	0.848
F2	149.278^Ba^ ± (25.474)	159.215^Bab^ ± (12.415)	172.378^Bb^ ± (11.560)	0.024
EQ	101.752^Aa^ ± (26.110)	103.447^Aa^ ± (20.156)	101.231^Aa^ ± (11.430)	0.968
AC	218.263^Ca^ ± (22.21)	232.183^Cab^ ± (14.536)	248.589^Cb^ ± (8.6384)	0.001
*p* value	<0.001	<0.001	<0.001	

In the same column, different superscript capital letters indicate statistically significant differences among the materials. In the same row, different superscript lowercase letters indicate statistically significant differences among the radiation doses within the material (*p* < 0.05).

**Fig. 3:**
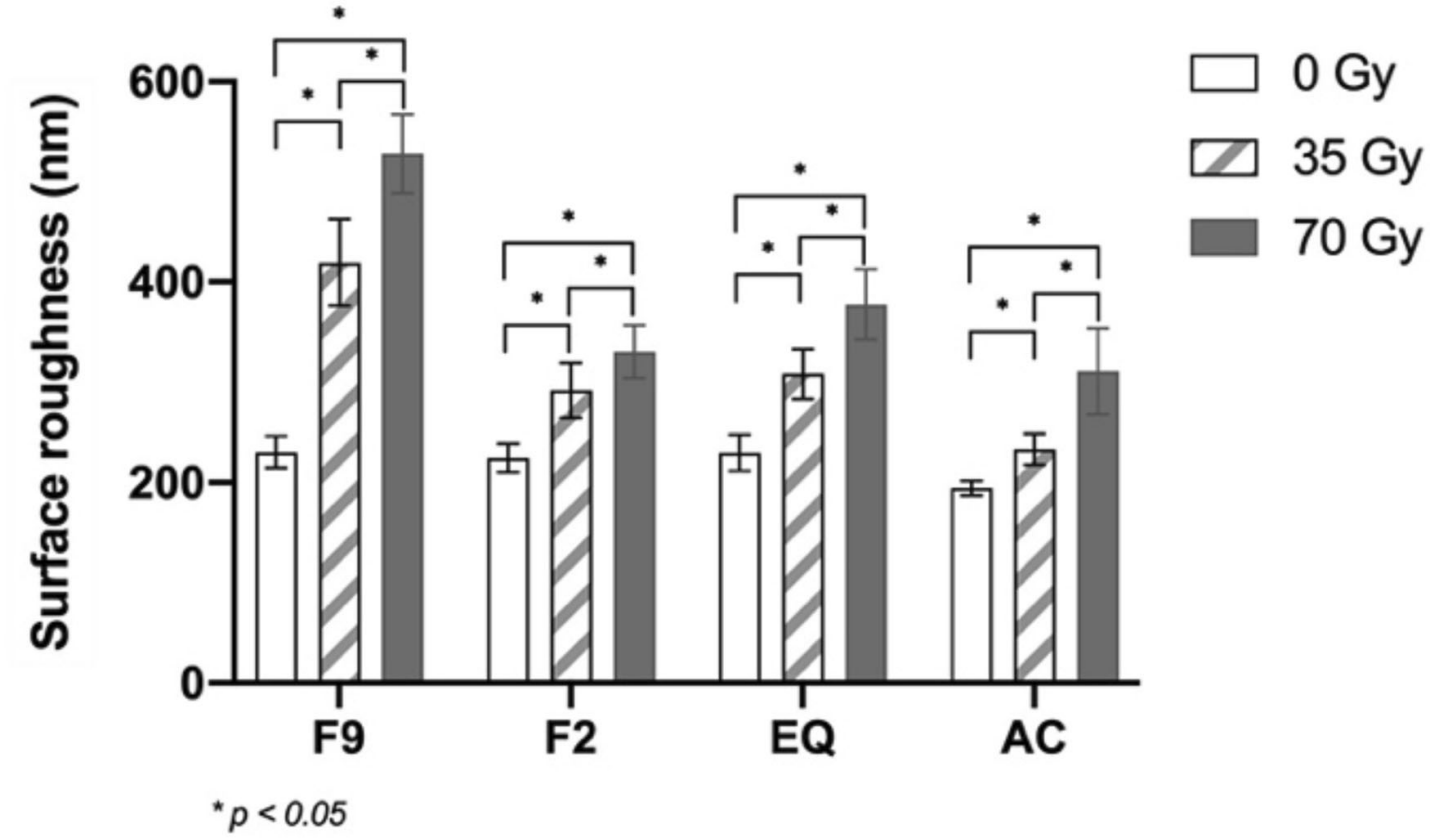
Mean surface roughness (nm) at different radiation doses. Asterisks (*) indicate significant differences (*p* < 0.05). The vertical bars indicate standard deviation.

The surface roughness test revealed that F9 exhibited the highest surface roughness, while AC demonstrated the lowest surface roughness when comparing materials exposed to the same radiation dose. Among all groups, the 70 Gy group had a significantly greater surface roughness than the other groups within the same materials (*p* < 0.001) (Fig. 3).

**Fig. 4:**
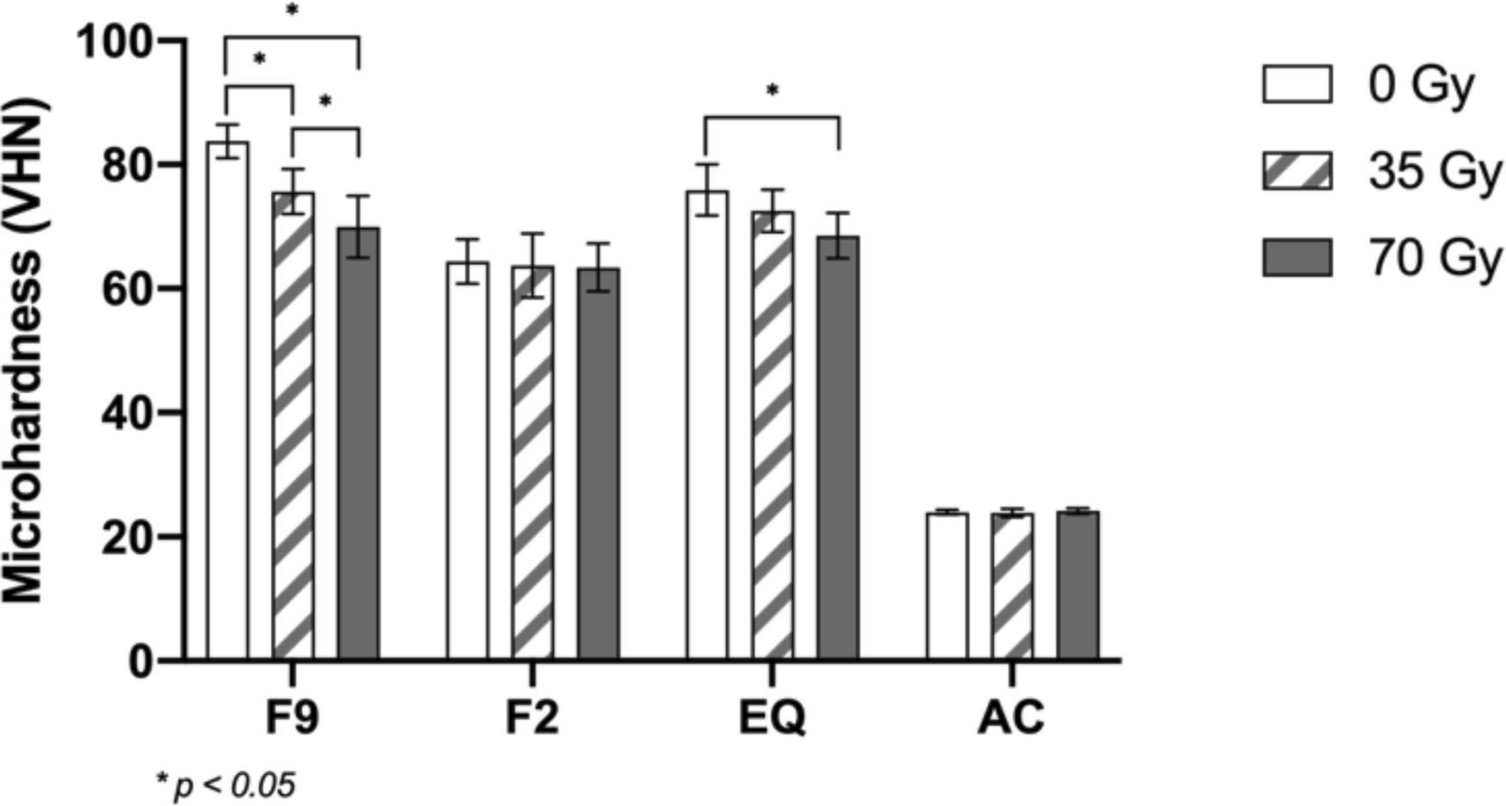
Mean microhardness (VHN) at different radiation doses. Asterisks (*) indicate significant differences (*p* < 0.05). The vertical bars indicate standard deviation.

The Vickers microhardness test also demonstrated that F9 had the highest microhardness, while AC had the significantly lowest microhardness among all groups at each radiation dose (*p* < 0.001). The microhardness in F9-70 and EQ-70 was significantly lower than those at baseline, but there were no significant differences in F2 and EQ among the radiation doses (Fig. 4).

**Fig. 5:**
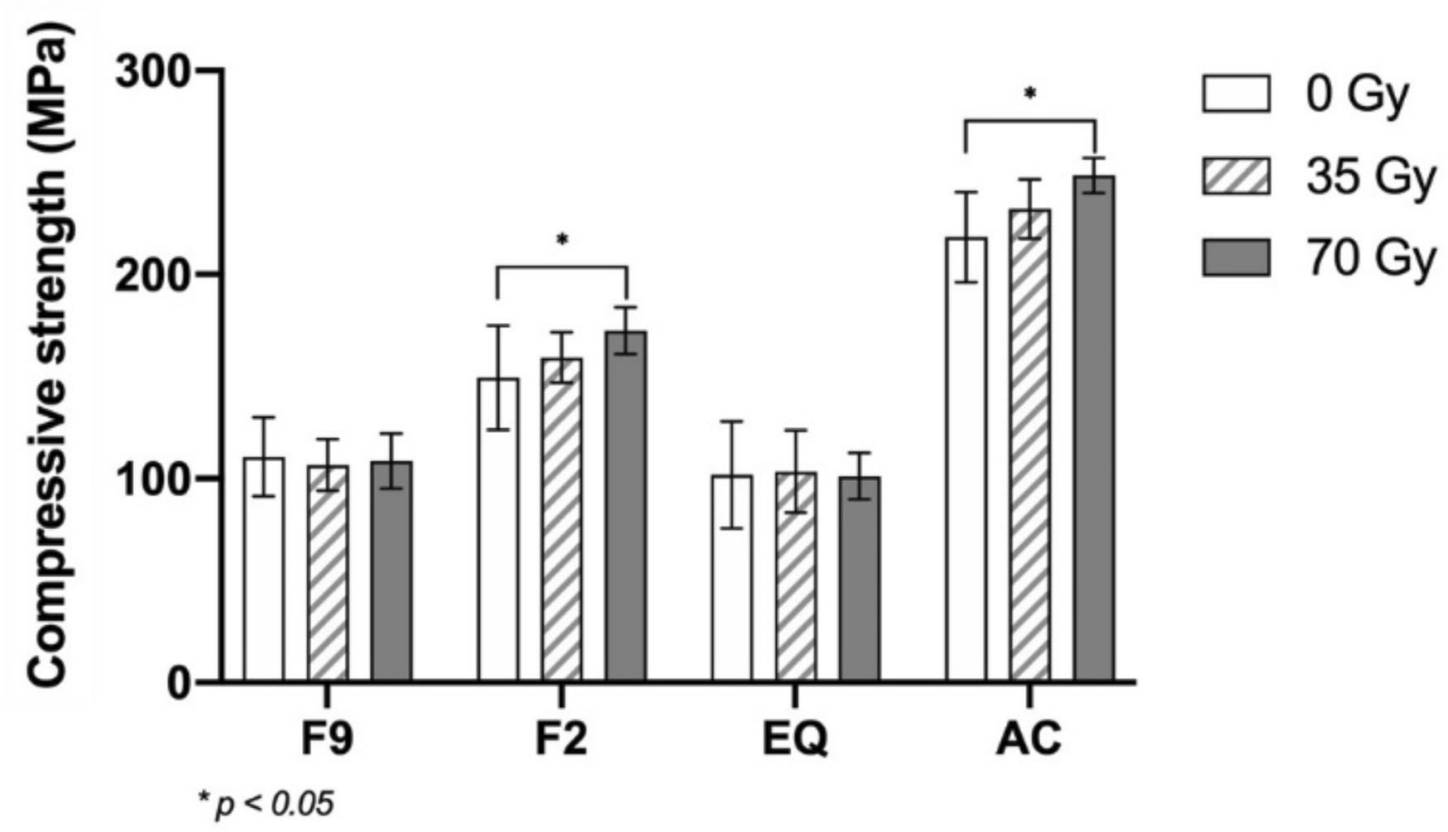
Mean compressive strength (MPa) at different radiation doses. Asterisks (*) indicate significant differences (*p* < 0.05). The vertical bars indicate standard deviation.

AC had the highest compressive strength, followed by F2. F2-70 and AC-70 had significantly greater compressive strengths than F2-0 and AC-0, respectively (*p* < 0.05), in contrast to F9-70 and EQ-70, which showed no significant difference form F9-0 and EQ-0 (*p* > 0.05) (Fig. 5).

**Fig. 6:**
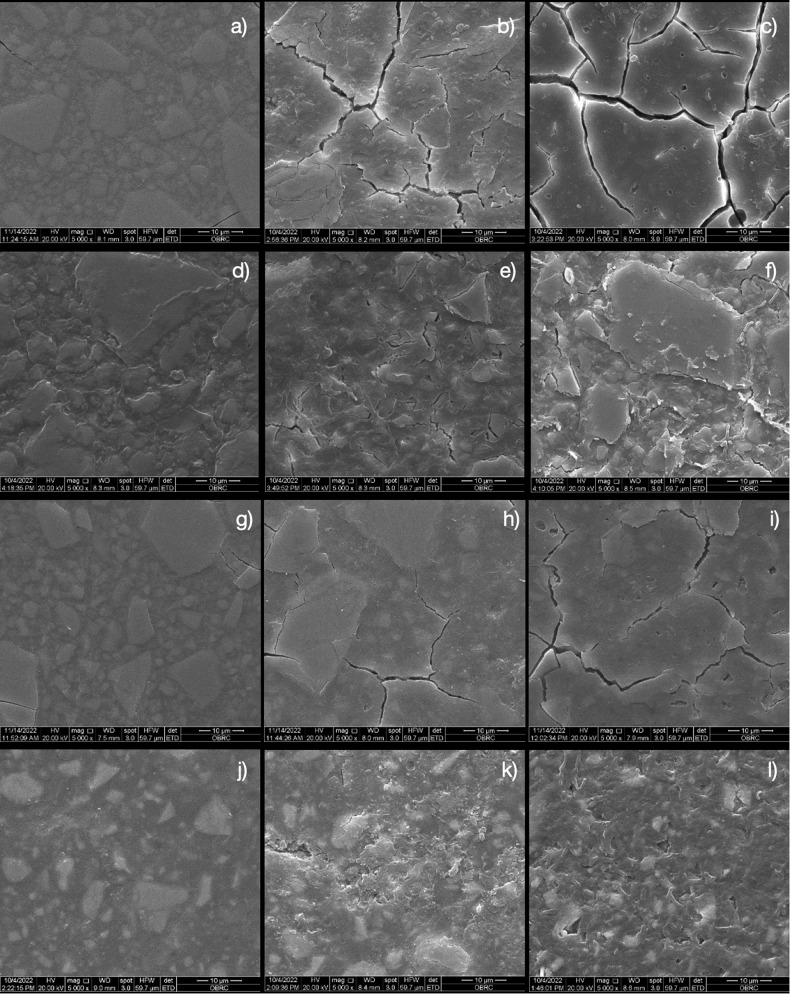
SEM photomicrographs demonstrating the surface of fluoride-releasing materials at 5000× magnification. **a** F9-0 (**b**) F9-35 (**c**) F9-70; **d** F2-0, (**e**) F2-35 (**f**) F2-70; **g** EQ-0, (**h**) EQ-35 (**i**) EQ-70; **j** AC-0, (**k**) AC-35 (**l**) AC-70.

SEM images of each group are shown in Fig. 6. More filler particles were exposed on the material surface at a higher radiation dose. F9-0 and EQ-0 showed no voids while F9-35 and EQ-35 exhibited intermittently scattered cracks and voids. Cracks could be clearly observed in the F9-70 and EQ-70 specimens. Compared with those in the F9 and EQ groups, the void and cracked lines in the F2 and AC groups were less pronounced at the same radiation dose. The chemical elements of the main component of each material are presented in Table 4. All tested materials were mainly composed of fluoride (F), calcium (Ca), aluminum (Al), silicon (Si), carbon (C), and oxygen (O). However, phosphorus (P) was detected only in F9, EQ, and AC, while sodium (Na) was detected only in F9 and EQ.

**Table 4:** Distribution of the chemical elements in the composition of fluoride-releasing materials in relative percentage by weight (wt%).

Groups	Chemical element (wt%)							
	F	Ca	Al	Na	P	Si	C	O
F9-0	6.63	0.48	11.93	2.03	1.68	8.53	25.33	37.49
F9-35	7.43	2.57	12.39	1.36	1.44	8.36	23.74	37.93
F9-70	7.26	1.36	8.73	0.80	1.37	6.34	33.62	37.99
F2-0	4.88	0.38	9.33	-	-	9.29	32.41	33.29
F2-35	4.44	1.42	9.40	-	-	10.72	27.76	36.70
F2-70	4.87	1.98	7.96	-	-	8.60	34.65	32.87
EQ-0	11.13	0.93	11.18	1.50	1.74	9.99	16.19	33.59
EQ-35	9.48	1.50	10.70	1.34	1.50	9.72	19.35	38.42
EQ-70	9.41	0.74	11.88	1.39	1.64	9.25	20.20	35.42
AC-0	2.02	1.53	2.77	-	0.50	11.07	42.54	30.17
AC-35	2.46	1.83	2.91	-	0.63	10.24	43.41	30.11
AC-70	2.32	0.20	2.46	-	0.24	9.95	46.80	30.29

## Discussion

This study aimed to verify the effects of ionizing radiation at 0, 35, and 70 Gy on mechanical properties of fluoride-releasing materials. The result revealed that ionizing radiation promoted changes in surface roughness, Vickers microhardness, and compressive strength. Therefore, the null hypothesis was rejected.

The use of IMRT is the accepted method for treating head and neck patients, and the amount of radiation applied depends on the cancer stage and the goal of treatment, whether curative or palliative [14]. During radiotherapy, head and neck cancer patients typically receive a total radiation dose ranging from 14 to 70 Gy which is usually applied in daily fractions of 1.8 to 2 Gy over seven weeks [15]. Therefore, 35 and 70 Gy fractional radiation were used to simulate radiotherapy in the present study.

In this study, a high viscosity GIC (F9) and glass hybrid material (EQ) are categorized as conventional GICs since they are produced by an acid-base reaction, while RMGIC (F2) retains the acid-base reaction with the addition of radical polymerization of the methacrylate monomer. Bioactive composite (AC) has been introduced by the manufacturer as a resin composite with the ability to induce apatite formation from a physiological solution. However, a study by Tiskaya et.al. revealed that AC could release Ca, P, and F ions but the amount of ions release was relatively low, and apatite formation was not observed. This indicated that the glass components in AC were fluoro-alumino-silicate glasses that could degrade in an acidic environment, resembling the glasses utilized in GIC [16]. Moreover, the resin polymerization reaction of AC is activated by chemopolymerization activators, followed by photopolymerization and acid-base reaction [3]. Therefore, AC is considered to have properties similar to RMGIC.

The surface roughness of fluoride-releasing materials significantly increased after exposure to ionizing radiation, which was in accordance with the findings of Brandeburski et al. The results of the studies showed an increase in surface roughness after fractional radiation at doses of 70.2 Gy [11]. Additional studies had reported that ionizing radiation induced changes in the surface roughness of other fluoride-releasing materials, such as Amalgomer CR (ceramic-reinforced GIC) and Zirconomer (zirconia-reinforced GIC) [17]. This hypothesis suggested that ionizing radiation interacted with the chemical components within the materials, potentially amplifying the radiation effects. However, there has not been a comprehensive explanation of the mechanism for the increase in surface roughness. It is imperative to conduct additional research to explore this phenomenon.

The authors speculated that the increase in surface roughness was probably influenced by water radiolysis, which was the process of water molecules dissociating due to ionizing radiation [18]. Water radiolysis generates various reactive species that potentially interfere with the bonding between the fillers and the matrix, leading to an increase in the surface roughness. The setting reaction of GIC extended several months due to its acid-base reaction. The water diffusion coefficient rate within the GIC materials took 4–6 weeks to reach completion during the maturation process [19]. The water that initiates radiolysis could originate from the material itself and the water storage environment. Due to the higher water content in conventional GIC than in RMGIC, more pronounced surface irregularities were observed in conventional GIC than in RMGIC.

The results of the microhardness test from this study are consistent with those of the research conducted by Amorim et al. who reported a reduction in the Vickers microhardness of conventional GIC after radiation whereas RMGIC value were significantly similar [20]. The hardness of the material was influenced by the number of exposed fillers, the bond stability between the fillers and the matrix, and the surface irregularity. The conventional GIC powder also contained non - silanized fluoroalumiosilicate filler (FASF) and lacked methacrylate monomers. Consequently, less exposure of filler particles could occur if the numerous fillers detach from the material surface, resulting in a decrease in microhardness after radiation [21]. In contrast, RMGIC contains silanized FASF and methacrylate monomers, which increase the stability of the bond between the fillers and the matrix [22].

The decision to conduct a compressive strength test according to ISO9917-1 and ISO9917-2 was made in this study because it is suitable for brittle materials including conventional GIC. The heightened compressive strength in RMGIC could be attributed to the post-cure reactions typical of resin-containing materials. This is possibly because ionizing radiation was absorbed by the inorganic filler particles as well as radiosensitive chemical groups. This radiation then propagates through the resin matrix, leading to excitation and ionization of the matrix and resulting in the generation of reactive species. Therefore, the crosslinking among the polymerized chains of the resin matrix increased. These radicals also gradually converted double bonds over time, resulting in a higher degree of conversion [23] and compressive strength [24]. These results are in agreement with the study by Vaishnavi, who reported that the mechanical properties of radiated resin contained materials further optimized the material properties [25]. An increase in compressive strength was observed only for F2-70 and AC-70. Nevertheless, the compressive strength remained relatively consistent in F9 and EQ due to the absence of a polymerization process in conventional GIC.

The SEM findings were correlated with the surface roughness. Exposure to ionizing radiation results in the dissolution of the material surface, leading to the emergence of subsurface filler particles distributed beneath the surface layer [20]. The surface irregularities of the materials influenced bacterial adhesion and biofilm formation, which could contribute to increased inflammation of the periodontal tissue [11].

EDS analysis revealed no substantial differences in the primary chemical components after radiation. This analysis has limitations in detecting low molar mass elements and was conducted on the surface at a depth of approximately 1 μm. Therefore, this approach did not provide an exact representation of the chemical compositions of the materials [17]. The present study showed that, compared with RMGIC, conventional GIC had a greater percentage of fluoride [26, 27]. The lower fluoride component in RMGIC could be attributed to the fact that the polymerized resin matrix restricted ion exchange with the external environment [26].

Based on this study, RMGIC appeared to be less susceptible to the effect of ionizing radiation on the surface roughness and microhardness with increasing compressive strength. Therefore, RMGIC could be considered a preliminary restorative material before the initiation of radiotherapy.

The coating agent was not applied after specimen preparation in any of the groups since it would be dislodged after clinical use due to the masticatory force and brushing process [28]. Consequently, our study was intentionally designed to assess the performance of fluoride-releasing materials without the influence of additional coatings. The specimens in this study were stored in distilled water since most HNRT patients typically experience hyposalivation to simulate the worst-case scenario. It has been reported that the parotid gland can reduce salivary function at a mean dose exceeding 20–25 Gy after radiation [29]. Moreover, an increased risk of oral candidiasis was reported in HNRT patients due to a reduction in the salivary flow rate [30]. Changes in microbial colonies in the oral cavity may impact the mechanical properties and longevity of restorative materials. Therefore, further research is needed to gather additional information in this regard.

## Conclusion

According to the results of this study, it is concluded that the exposure to ionizing radiation negatively affected certain mechanical properties of the fluoride-releasing materials, particularly conventional glass ionomer cement.

## Data Availability

The data supporting the findings of this study can be obtained from the corresponding author, DN, upon reasonable request.

## References

[1] Sung H, Ferlay J, Siegel RL, Laversanne M, Soerjomataram I, Jemal A (2021). Global Cancer Statistics 2020: GLOBOCAN estimates of incidence and mortality worldwide for 36 Cancers in 185 Countries. CA Cancer J. Clin..

[2] Sroussi HY, Epstein JB, Bensadoun RJ, Saunders DP, Lalla RV, Migliorati CA (2017). Common oral complications of head and neck cancer radiation therapy: mucositis, infections, saliva change, fibrosis, sensory dysfunctions, dental caries, periodontal disease, and osteoradionecrosis. Cancer Med.

[3] Francois P, Fouquet V, Attal J-P, Dursun E (2020). Commercially available fluoride-releasing restorative materials: a review and a proposal for classification. Materials.

[4] Mickenautsch S, Yengopal V (2016). Caries-preventive effect of high-viscosity glass ionomer and resin-based fissure sealants on permanent teeth: a systematic review of clinical trials. PLoS One.

[5] Joshi G, Heiss M (2021). Glass-hybrid technology for long-term restorations. Compend Contin. Educ. Dent..

[6] Menezes-Silva R, Cabral RN, Pascotto RC, Borges AFS, Martins CC, Navarro MFL (2019). Mechanical and optical properties of conventional restorative glass-ionomer cements - a systematic review. J. Appl Oral. Sci..

[7] Viero FL, Boscolo FN, Demarco FF, Faot F (2011). Effect of radiotherapy on the hardness and surface roughness of two composite resins. Gen. Dent..

[8] von Fraunhofer JA, Curtis P, Sharma S, Farman AG (1989). The effects of gamma radiation on the properties of composite restorative resins. J. Dent..

[9] Cruz ADD, Sinhoreti MAC, Ambrosano GM, Rastelli ANDS, Bagnato VS, Bóscolo FN (2008). Effect of therapeutic dose X rays on mechanical and chemical properties of esthetic dental materials. Mater. Res.

[10] Catelan A, Padilha A, Salzedas L, Coclete GA, dos Santos PH (2008). Effect of radiotherapy on the radiopacity and flexural strength of a composite resin. Acta Odontol. Latinoam..

[11] Brandeburski SBN, Della Bona A (2018). Effect of ionizing radiation on properties of restorative materials. Dent. Mater..

[12] ISO 9917-1. Dentistry - Water-based cements - Part 1 Acid-base cements. International Standard Organization, 2007.

[13] ISO 9917-2. Dentistry - Water-based cements - Part 2 Resin-modified cements. International Standard Organization, 2010.

[14] Kaur J, Mohanti BK (2011). Transition from curative to palliative care in cancer. Indian J. Palliat. Care.

[15] Radiologists RCo. Radiotherapy Dose Fractionation. Third edition. Royal College of Radiologists; London, UK Chapter 6 Head and neck cancer. 2019.

[16] Tiskaya M, Al-Eesa NA, Wong FSL, Hill RG (2019). Characterization of the bioactivity of two commercial composites. Dent. Mater..

[17] Ugurlu M, Ozkan EE, Ozseven A (2020). The effect of ionizing radiation on properties of fluoride-releasing restorative materials. Braz. Oral. Res.

[18] Dzaugis ME, Spivack AJ, D’Hondt S (2015). A quantitative model of water radiolysis and chemical production rates near radionuclide-containing solids. Radiat. Phys. Chem.

[19] Nicholson JW (2018). Maturation processes in glass-ionomer dental cements. Acta Biomater. Odontol. Scand..

[20] de Amorim DMG, Veríssimo AH, Ribeiro AKC, de Assunção ESRO, de Assunção IV, Caldas M (2021). Effects of ionizing radiation on surface properties of current restorative dental materials. J. Mater. Sci. Mater. Med.

[21] Kundie FAC, Muchtar A, Ahmad ZA (2018). Effects of filler size on the mechanical properties of polymer-filled dental composites. A review of recent developments. J. Phys. Sci..

[22] Pratap B, Gupta RK, Bhardwaj B, Nag M (2019). Resin based restorative dental materials: characteristics and future perspectives. Jpn Dent. Sci. Rev..

[23] Zissis A, Yannikakis S, Polyzois G, Harrison A (2008). A long term study on residual monomer release from denture materials. Eur. J. Prosthodont Restor. Dent..

[24] Taher RM, Moharam LM, Amin AE, Zaazou MH, El-Askary FS, Ibrahim MN (2021). The effect of radiation exposure and storage time on the degree of conversion and flexural strength of different resin composites. Bull. Natl Res. Cent..

[25] Vaishnavi C, Kavitha S, Narayanan LL (2010). Comparison of the fracture toughness and wear resistance of indirect composites cured by conventional post curing methods and electron beam irradiation. J. Conserv Dent..

[26] Nigam AG, Jaiswal J, Murthy R, Pandey R (2009). Estimation of fluoride release from various dental materials in different media-an in vitro study. Int J. Clin. Pediatr. Dent..

[27] Mousavinasab SM, Meyers I (2009). Fluoride release by glass ionomer cements, compomer and giomer. Dent. Res J.

[28] Habib SI, Yassen AA, Bayoumi RE (2021). Influence of nanocoats on the physicomechanical properties and microleakage of bulk-fill and resin-modified glass ionomer cements: an in vitro study. J. Contemp. Dent. Pract..

[29] Brodin NP, Tomé WA (2018). Revisiting the dose constraints for head and neck OARs in the current era of IMRT. Oral. Oncol..

[30] Tarapan S, Matangkasombut O, Trachootham D, Sattabanasuk V, Talungchit S, Paemuang W (2019). Oral Candida colonization in xerostomic postradiotherapy head and neck cancer patients. Oral. Dis..

